# THE FUTURE OF AGING RESEARCH: OPPORTUNITIES AND CHALLENGES FROM GLOBAL AGING SURVEYS

**DOI:** 10.1093/geroni/igae098.1061

**Published:** 2024-12-31

**Authors:** Jinkook Lee

**Affiliations:** University of Southern California, Los Angeles, California, United States

## Abstract

Jinkook Lee is the Director of the Program on Global Aging, Health, and Policy at USC. Dr. Lee advocates for the development of robust data and research infrastructure to better understand aging populations. She has been involved in initiatives like the Harmonized Cognitive Assessment Protocol (HCAP) and is the architect of the Gateway to Global Aging Data, a platform that facilitates cross-national and longitudinal research on aging by providing access to harmonized data from various international studies of older adults. The Gateway serves as a valuable resource for researchers, policymakers, and academics interested in comparing aging-related outcomes across different countries and over time. In this BSS Presidential Symposium, Dr. Lee will describe the challenges and opportunities associated with research on aging around the globe. She will discuss the shift from a focus on aging as being free of disease to a focus on multidimensional aspects of healthy aging, utilizing a life-course framework and capturing multiple aspects of exposure to deepen our understanding of the aging process, and the challenges of environmental hazards for our rapidly aging world. Dr. Lee’s presentation will provide useful background for thinking about how the next decade of research using studies from around the world will advance research on the aging process and aging populations around the world.

